# Renal Denervation Suppresses the Inducibility of Atrial Fibrillation in a Rabbit Model for Atrial Fibrosis

**DOI:** 10.1371/journal.pone.0160634

**Published:** 2016-08-16

**Authors:** Yong Wei, Juan Xu, Genqing Zhou, Songwen Chen, Ping Ouyang, Shaowen Liu

**Affiliations:** 1 Department of cardiology, Shanghai Songjiang Central Hospital, Shanghai, 201600, China; 2 Department of cardiology, Shanghai First People’s Hospital, School of Medicine, Shanghai Jiao Tong University, Shanghai, 200800, China; University Medical Center Utrecht, NETHERLANDS

## Abstract

Renal denervation (RD) was reported to reduce the susceptibility of atrial fibrillation (AF), but the underlying mechanism has not been well understood. This study was performed to investigate the effect of RD on the inducibility of AF in a rabbit model for atrial fibrosis and to explore the potential mechanisms. Thirty-five rabbits were randomly assigned into sham-operated group (n = 12), abdominal aortic constriction (AAC) group (n = 12) and AAC with RD (AAC-RD) group (n = 11). The incidence of AF induced by burst pacing in atriums was determined. Blood was collected to measure the levels of rennin, angiotensin II and aldosterone. Atrial samples were preserved to evaluate protein and gene expression of collagen, connective tissue growth factor (CTGF) and transforming growth factor-β1 (TGF-β1). Our data suggested cardiac structure remodeling and atrial fibrosis were successfully induced by AAC. Compared with the AAC group, the AAC-RD rabbits had smaller ascending aortic diameter and left ventricular end-systolic diameter. For burst pacing at the left atrium (LA), AF was induced in two of the 12 rabbits in the sham-operated group, 10 of the 12 rabbits in the AAC group, and 2 of the 11 rabbits in the AAC-RD group, with great difference among the three groups (P = 0.001). The percentage of LA burst stimulations with induced AF achieved 47.2% in the AAC group, which was higher than those in both the AAC-RD (12.1%) and the Sham-operated (5.6%) groups. Significantly increasing intercellular space in the AAC group (P<0.001) compared with the sham-operated rabbits. RD clearly decreased the volume fraction of collagen in LA and right atrium compared with that of the AAC group (P< 0.01). AAC-induced elevation of collagen I, CTGF and TGF-β1 was suppressed by RD. In conclusion, RD suppressed the inducibility of AF in a rabbit model for pressure associated atrial fibrosis, potentially by modulating renin-angiotensin-aldosterone system and decreasing pro-fibrotic factors.

## Introduction

Atrial fibrillation (AF) is one of the most common arrhythmia and associated with high morbidity and mortality, but its pathogenesis is insufficiently understood. Pulmonary vein triggers, micro-reentrant circuits and atrial remodeling are regarded as the key pathophysiological basis. It’s indicated autonomic nervous system also plays an important role in the occurrence and maintenance of AF [1,2,3]. Pokushalov E, et al. demonstrated renal denervation (RD) reduced AF recurrences when combined with pulmonary vein isolation (PVI) [4]. Animal experiments showed episodes of AF were decreased by renal sympathetic denervation during both 7-hour and prolonged rapid atrial pacing [5,6]. However, the potential mechanisms for RD to reduce the susceptibility of AF have not been clearly understood so far.

For RD was reported to reduce sympathetic activity [4], we hypothesized that RD might alter the Renin-Angiotensin-Aldosterone System (RAAS) activation via regulating the sympathetic nervous system, subsequently inhibiting atrial fibrosis and reducing the substrate of AF. This study was performed to verify that RD could suppress the inducibility of AF in a rabbit model of atrial fibrosis induced by abdominal aortic constriction (AAC), potentially by mediating RAAS.

## Methods

### Ethics Statement

All animal protocols in this study were approved by the Animal Care and Use Committee, Research Institute of Medicine, Shanghai Jiao Tong University, in accordance with the guide for the Care and Use of Laboratory Animals published by the National Institutes of Health (Publication No. 85–23, revised 1996). All efforts were paid to minimize animal suffering. We euthanized the animals with an overdose of anesthesia when they met un-healing incision with serious infection. There were three rabbits undergoing euthanization. The health of rabbits was monitored everyday in the first week after operation and thereafter one time per week. There was only one unexpected death, whose cause was acute intestinal obstruction happening in the second day after operation. Preoperative sedation and postoperative analgesia were applied to minimized animal suffering and distress.

### Animal model for atrial fibrosis and renal denervation

An animal model of atrial fibrosis, established as an appropriate experimental model of AF, was induced by pressure overload via AAC [7]. Thirty-nine young male New Zealand White rabbits weighing 2300 to 2600 grams were randomly assigned to three groups as the sham-operated group, the AAC group and the AAC-RD group.

The procedures of AAC were as follows. The studied rabbits were anesthetized with sodium pentobarbital (50 mg/kg IV). After opening the abdomen, the abdominal aorta below renal arteries was released from the connective tissue, and a bent 7-gauge needle was placed next to the abdominal aorta. The suture was securely tied around the needle and the aorta. After ligation, the needle was quickly removed. RD was accomplished by using a surgical-pharmacological procedure adapted as previously described [8]. Mechanical denervation was performed by carefully stripping all visible nerves along the renal arteries and veins from the aorta to the hilum of the kidney. Chemical denervation was also performed by quickly painting the renal artery with 20% phenol in absolute ethanol. Then, the artery was washed with isotonic saline. Rabbits in the AAC group underwent AAC only. And rabbits in the AAC-RD group received both AAC and RD. For sham-operated rabbits, the surgical procedure was the same, but the abdominal aorta was not ligated and the nerves along with the renal arteries were left intact. Four months after the surgical procedure, Color Doppler Ultrasound was utilized to inspect the heart, the kidneys and the abdominal aorta. Such parameters as ascending aortic diameter (AAD), left atrium diameter (LAD), left ventricular end-diastolic diameter (LVEDD), left ventricular end-systolic diameter (LVESD), inter-ventricular septal thickness (IVST), left ventricular posterior wall thickness (LVPWT), left ventricular ejection factor (LVEF), left renal artery blood flow velocity (LRABFV), left renal artery diameter (LRAD), right renal artery blood flow velocity (RRABFV) and right renal artery diameter (RRAD) were recorded. After all protocols, the final sample size was: Sham-operated n = 12, AAC n = 12 and RD-AAC n = 11.

### Electrophysiological recordings

The final experiment of animals was performed four months after the surgical procedure. After the rabbits were anesthetized and got normal breathing, thoracotomy was performed. The pericardium was opened and the heart was clearly explored. The tips of two electrodes were directed to the left and the right atriums respectively. The surface electrocardiograms and electric signals arising from the above-mentioned electrodes were simultaneously recorded. After ten minutes of stabilization, a series of electrophysiological tests were initiated. Rapid S_1_S_1_ (50 ms cycle length) programmed stimuli were adopted to induce AF. The duration of S_1_S_1_ stimulation lasted for one second. AF was defined as irregular atrial rates faster than 500 bpm, lasting over one second. AF inducibility was repeated for 3 times by burst pacing at each atrium.

### Masson staining

The atrial tissue samples were fixed with 4% paraformaldehyde in phosphate-buffered saline for 24 h, and then were subjected to alcoholic dehydration and embedded in paraffin. 4-μm thick sections were sliced, which underwent Masson’s trichrome staining to highlight collagen fibers. Six random images were obtained from each slide and collagen volume fraction (CVF) was determined by Image Pro Plus software. The mean of the six CVF values was calculated as the final CVF of a slice.

### ELISA

Five millilitres of venous blood was collected in EDTA vacutainers and centrifuged at 2,310×g for 10 min at 4°C. The separated plasma was stored at −80°C. Plasma levels of renin, Ang II and aldosterone were determined by ELISA.

### Western Blotting

The atrial tissue samples were washed by phosphate buffered saline (PBS) and homogenized in RIPA solubilization buffer containing a 1:100 dilution of protease inhibitor (Beyotime Biotechnology). Protein concentrations were determined by BCA protein assay (Beyotime Biotechnology). After equal protein mixtures were separated on SDS-PAGE, gels were blotted to polyvinylidene difluoride membranes and the membranes were incubated in Tris-buffered saline containing 0.1% Tween 20 (TBST) with 5% milk for 1 hour at room temperature. Then, the membranes were incubated overnight with primary antibody including anti-TGF-β1 (1:200, LifeSpan BioSciences), anti-CTGF (1:200, Abbiotec) and anti-collagen (1:200, Abcam). Anti-GAPDH (1:5000, Proteintech) was used as an internal control. After washed by TBST, the blots were incubated with horseradish peroxidase-conjugated secondary antibodies (1:3000, Jackson) for 2 hours at room temperature.Proteins were visualized by enhanced chemiluminescence (Millipore), and images were obtained by exposure to films. The bands were scanned and quantified using (GE-ImageQuant-LAS-4000) imaging software. All the quantifications of bands were normalized by the corresponding value of GAPDH.

### Data analysis

Continuous data were given as mean±SEM. To compare 2 groups, an unpaired Student’s t-test was used. For multiple comparisons among three groups, one-way ANOVA was used followed by LSD test. Pearson’s chi-square test was applied to analyze count data. Differences were considered significant at P<0.05. For all statistical calculations software SPSS 13.0 was used.

## Results

### General characteristics of studied animals

**Fig 1:**
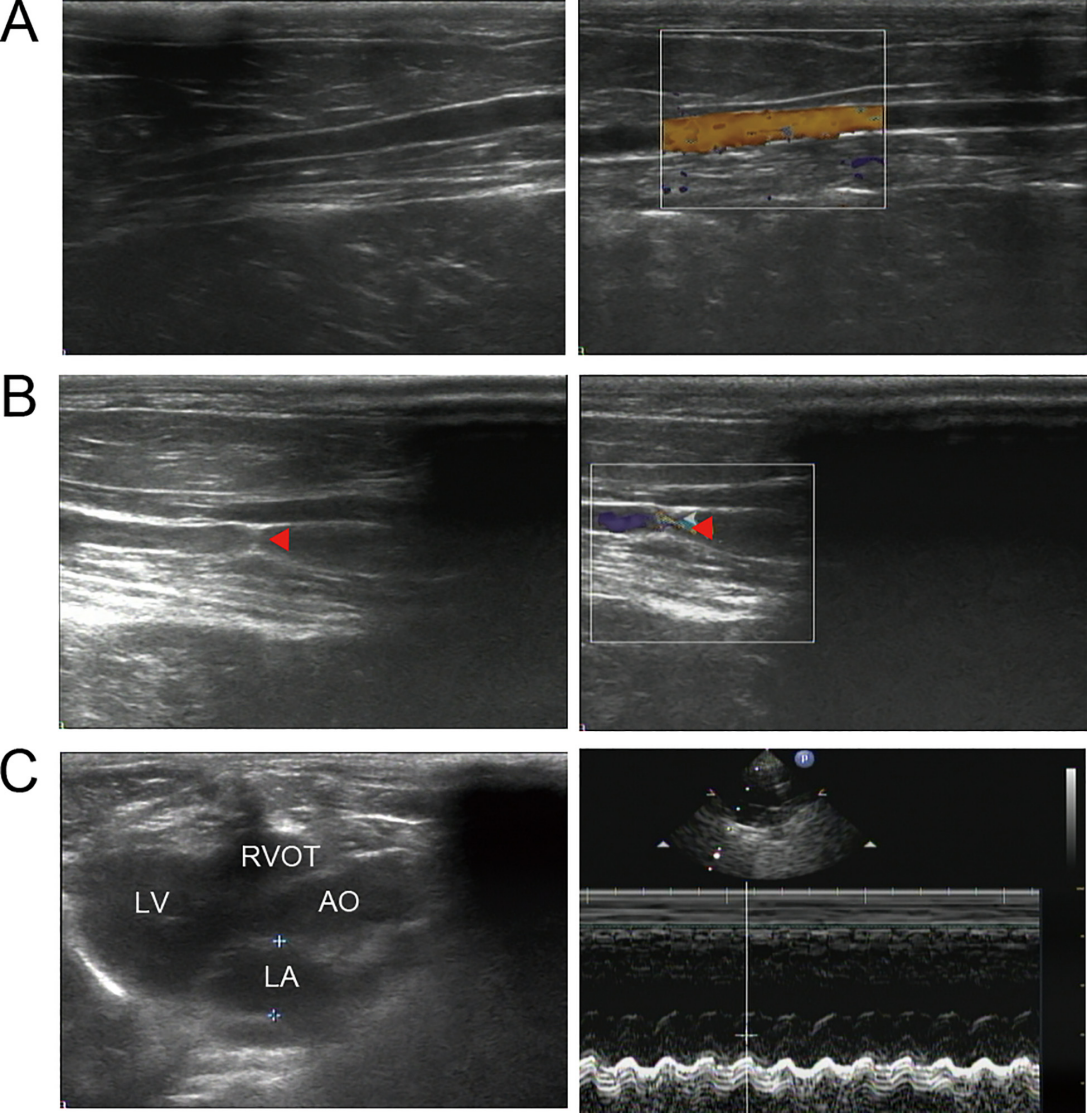
Color Doppler Ultrasound evaluation of abdominal aortic stenosis and cardiac structure of studied animals. The gray-scale sonographic appearance (the left) and the color doppler flow imaging (the right) of the abdominal aorta for sham-operated (A) and AAC (B) rabbits. Red triangle indicated the position of the abdominal aortic stenosis. (C) Representative echocardiographic image with two-dimensional parasternal long-axis view and M-mode echocardiogram for a studied rabbit. AAC, abdominal aortic constriction; LV, left ventricle; LA, left atrium; AO, aorta; RVOT, right ventricular outflow tract.

After all protocols, the final sample size was 12, 12 and 11 for the sham-operated group, the AAC group and the RD-AAC group respectively. General characteristics of the studied animals were presented in Table 1 and Fig 1. Body weights were similar among the three groups at baseline and 4 months after abdominal operation. Abdominal aortic stenosis was successfully acquired by nearly 45% (Fig 1), which was comparable between the AAC and AAC-RD groups (Table 1). Rabbits in the AAC group had greater AAD, LVEDD, LVESD, IVST, and LVPWT than those in the sham-operated group, which indicated that AAC effectively induced cardiac remodeling. Compared with the AAC group, the AAC-RD rabbits had smaller AAD and LVESD. It indicated that RD could inhibit AAC-induced cardiac remodeling. In addition, neither AAC nor RD altered renal artery diameter, which showed the invasive procedures performed for RD didn’t induce renal artery stenosis. AAC increased renal artery blood flow velocity, which was further augmented by RD.

**Table 1:** General characteristics of studied animals.

	Sham-operated (n = 12)	AAC (n = 12)	AAC-RD (n = 11)
Primary BW (Kg)	2.44±0.08	2.51±0.27	2.4±0.26
BW in 4^th^ month (Kg)	3.29±0.18	3.3±0.19	3.32±0.23
AAD (mm)	8.55±0.12	8.92±0.46*	8.48±0.46^#^
LAD (mm)	9.63±1.17	10.5±0.84	9.81±1.1
LVEDD (mm)	11.78±1.24	13.55±1.27**	13.83±0.99^&&^
LVESD (mm)	7.05±0.9	8.33±0.70**	7.48±0.74^#^
IVST (mm)	2.12±0.3	2.55±0.12**	2.52±0.13^&&^
LVPWT (mm)	2.1±0.26	2.4±0.09**	2.46±0.05^&&^
LVEF (%)	75±3	76±5	74±1
LRABFV (cm/s)	35.33±2.23	40±3.3**	44.91±5.99^##^^,^^&&^
LRAD (mm)	1.88±0.25	1.98±0.15	2.09±0.08^&&^
LA-LK (mm)	37.33±1.87	36.67±1.87	37.27±2.49
SA-LK (mm)	17.33±3.94	18.17±1.4	18.18±1.66
CSA-LK (mm^2^)	648±155.18	668.33±86.65	681.45±106.7
RRABFV (cm/s)	34.5±2.61	40.5±3.71**	48.18±7.04^##^^,^^&&^
RRAD (mm)	1.95±0.22	2.13±0.21	2.02±0.18
LA-RK (mm)	37.83±1.75	38.33±1.44	36.18±1.4^##^^,^^&^
SA-RK (mm)	17.67±0.78	19.83±1.75**	18.36±0.5^##^
CSA-RK (mm^2^)	667.83±31.64	758.17±44.27**	664.73±37.69^##^
AAS (%)	-	46±4	44±5

The AAC group vs. the sham-operated group

* indicated P<0.05 and

** indicated P<0.01. The AAC-RD group vs. the AAC group

^#^ indicated P<0.05 and

^##^ indicated P<0.01. The AAC-RD group vs. the Sham-operated group

^&^ indicated P<0.05 and

^&&^ indicated P<0.01.

AAC, abdominal aortic constriction; AAC-RD, abdominal aortic constriction with renal denervation; BW, body weight; AAD, ascending aortic diameter; LAD, left atrium diameter; LVEDD, left ventricular end-diastolic diameter; LVESD, left ventricular end-systolic diameter; IVST, inter-ventricular septal thickness; LVPWT, left ventricular posterior wall thickness; LVEF, left ventricular ejection factor; LRABFV, left renal artery blood flow velocity; LRAD, left renal artery diameter; RRABFV, right renal artery blood flow velocity; RRAD, right renal artery diameter; LA-LK, long axis of the left kidney; SA-LK, short axis of the left kidney; CSA-LK, cross-sectional area of the left kidney; LA-RK, long axis of the right kidney; SA-RK, short axis of the right kidney; CSA-RK, cross-sectional area of the right kidney; AAS, abdominal aortic stenosis.

### Effects of RD on AF inducibility

**Fig 2:**
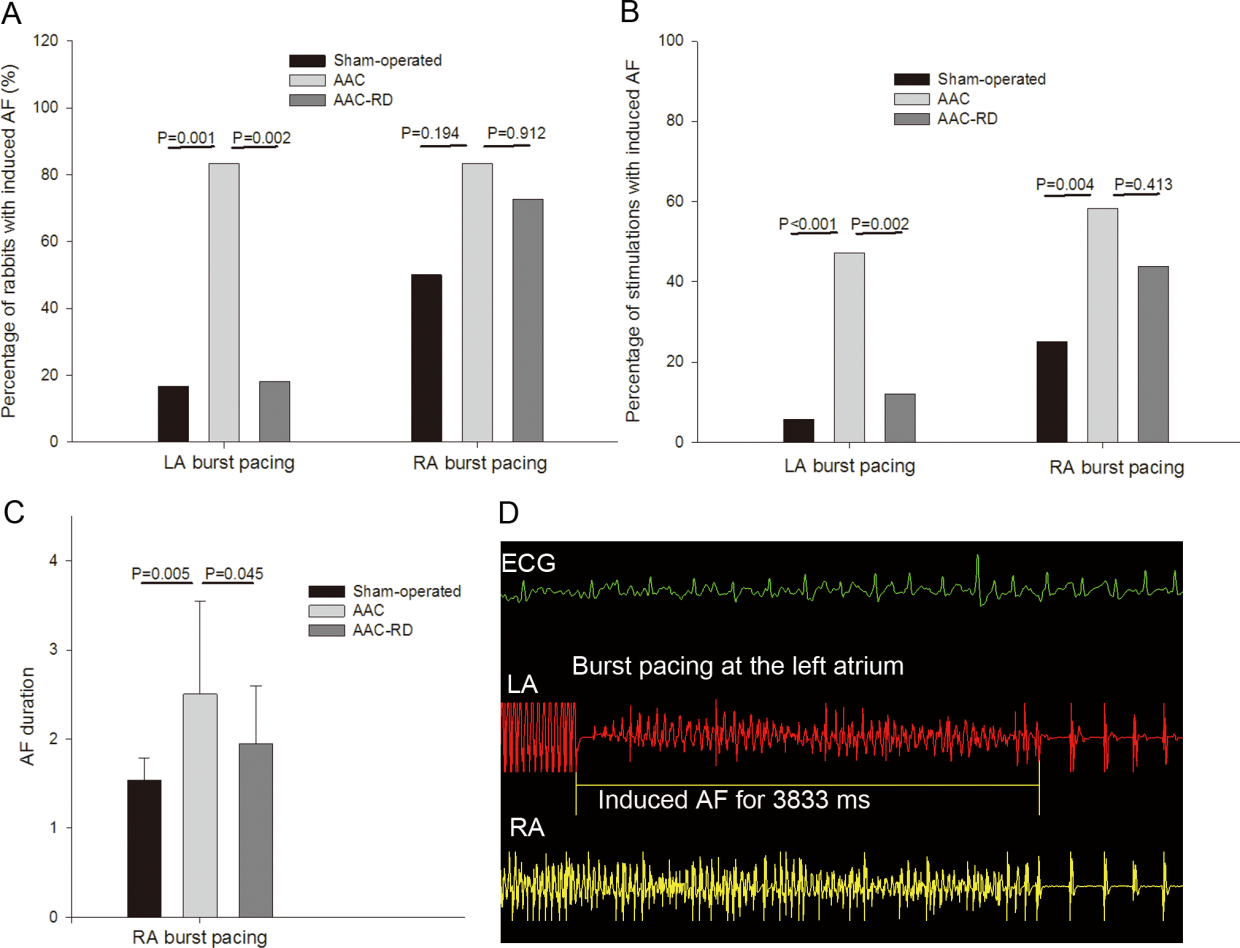
The inducibility of atrial fibrillation (AF) by burst atrial pacing. (A) The percentage of rabbits with induced AF after burst pacing in the left atrium (LA) and right atrium (RA). (B) The percentage of stimulations with induced AF after burst pacing in the LA and RA. (C) The duration of induced AF after burst pacing in the RA. (D) Representation of AF induced by atrial burst pacing. AAC, abdominal aortic constriction; abdominal aortic constriction with renal denervation (AAC-RD); ECG, electrocardiogram.

For LA burst pacing, AF was induced in two of the 12 rabbits in the sham-operated group, 10 of the 12 rabbits in the AAC group, and 2 of the 11 rabbits in the AAC-RD group, showing great difference among the three groups (P = 0.001) (Fig 2A). The percentage of LA burst stimulations with induced AF achieved 47.2% (3 burst stimulations per rabbit, 36 stimulations for 12 rabbits, 17 stimulations with induced AF, 17/36) in the AAC group, which was higher than those in both the AAC-RD (12.1%, 4/33) and the sham-operated (5.6%, 2/36) groups (P < 0.01) (Fig 2B). For RA burst pacing, AF inducibility was with no difference among the three groups (Fig 2A, Fig 2B), but ANOVA analysis showed great difference in the duration of induced AF (P < 0.05) (Fig 2C). Fig 2D showed a representative AF induced by burst pacing at the left atrium.

### Effect of RD on atrial fibrosis

**Fig 3:**
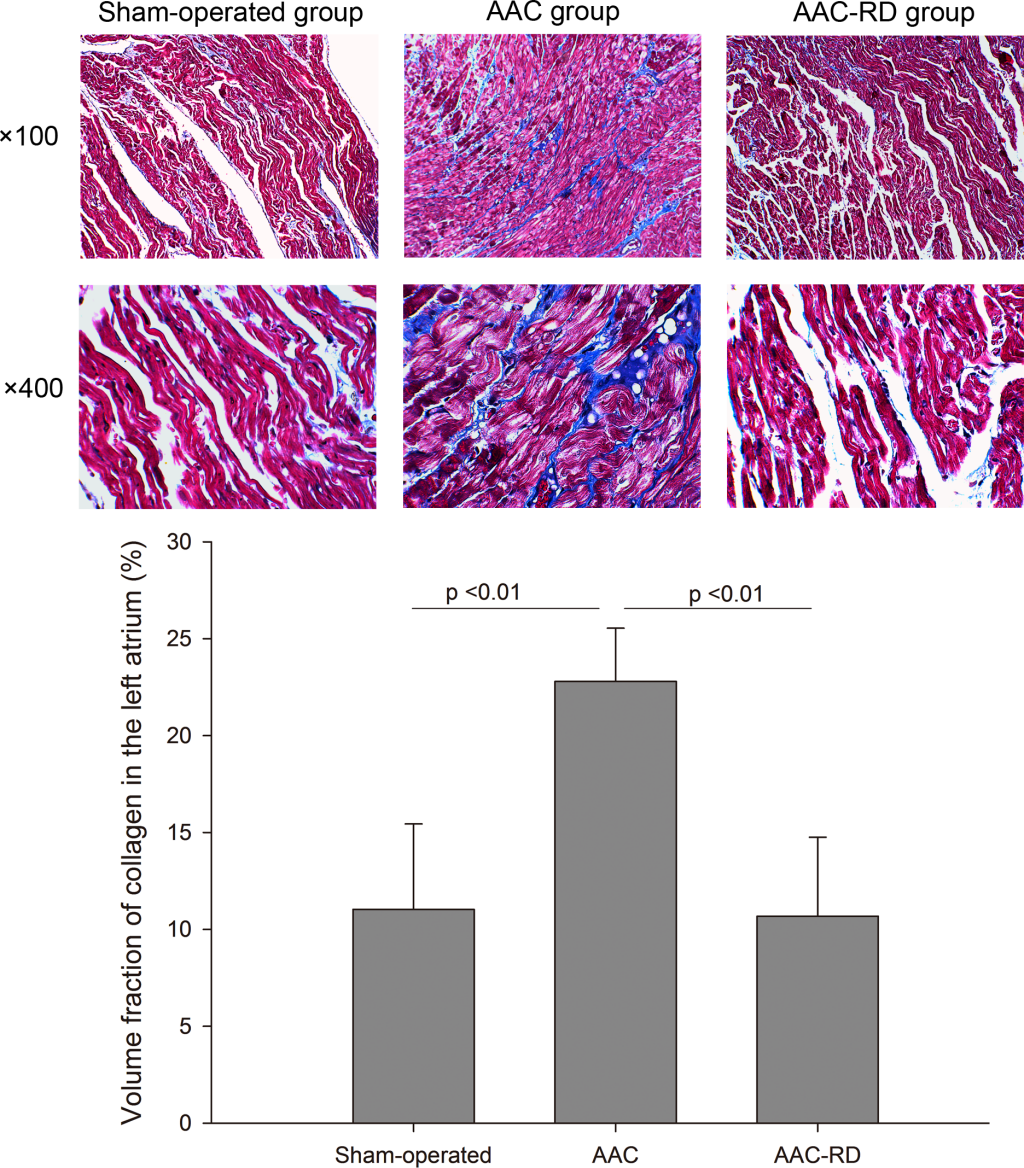
Effect of renal denervation on collagen fibers in the left atria detected by Masson staining. AAC, abdominal aortic constriction; AAC-RD, abdominal aortic constriction with renal denervation.

**Fig 4:**
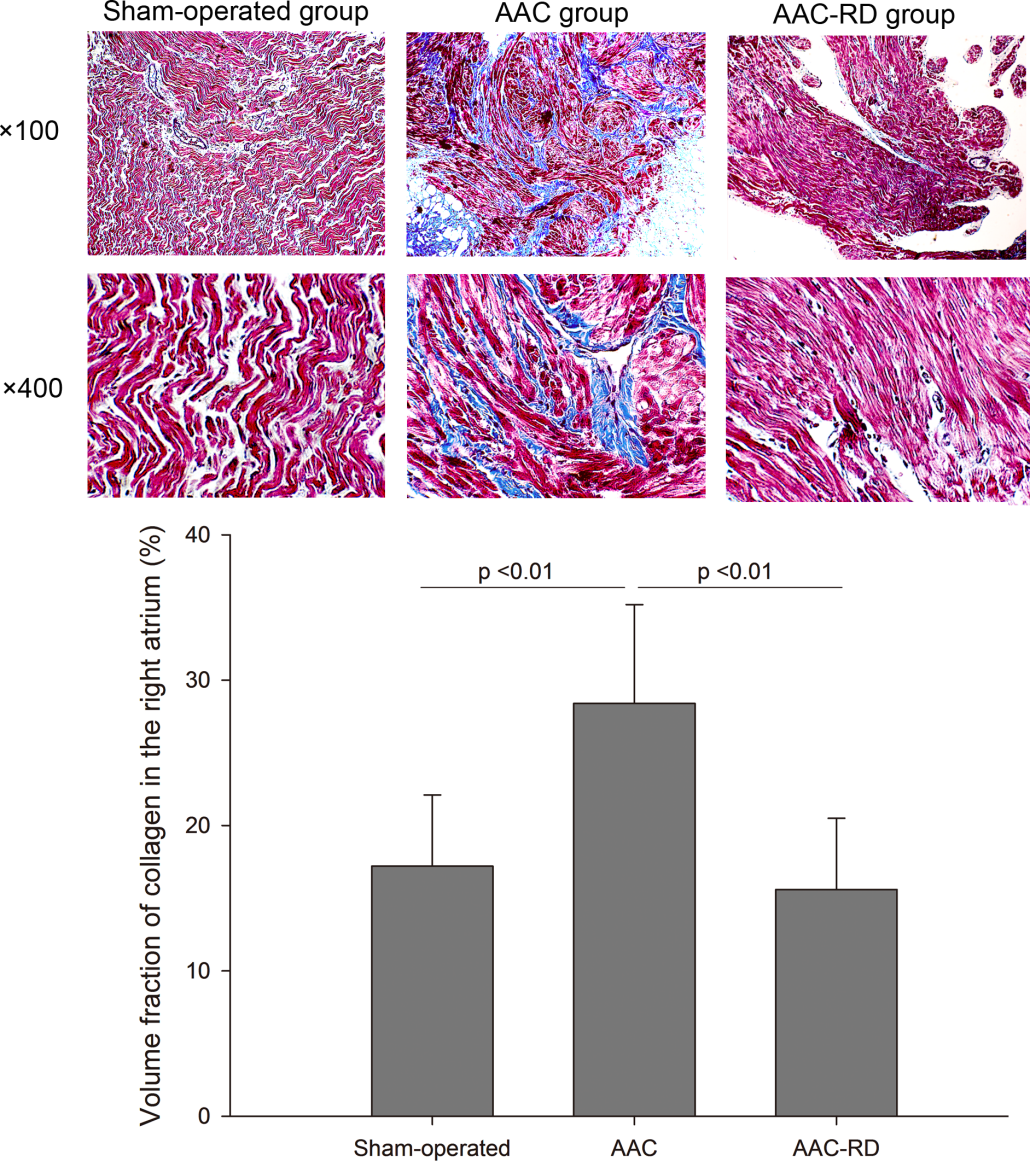
Effect of renal denervation on collagen fibers in the right atria detected by Masson staining. AAC, abdominal aortic constriction; AAC-RD, abdominal aortic constriction with renal denervation.

As shown in Fig 3, staining for collagen in the left atria showed a significantly increasing intercellular space in the AAC group compared with the sham-operated rabbits [Sham-operated vs. AAC, (11.03±4.41)% vs. (22.80±2.74)%, P < 0.01], meanwhile RD clearly decreased the volume fraction of collagen induced by AAC [AAC vs. AAC-RD, (22.80±2.74)% vs. (10.68±4.08)%, P < 0.01]. Staining for collagen in the right atria also indicated RD could suppress atrial fibrosis induced by AAC (Fig 4). Meanwhile, we also observed RD inhibited ventricular fibrosis induced by AAC (S1 Fig).

### Effect of RD on RAAS

**Fig 5:**
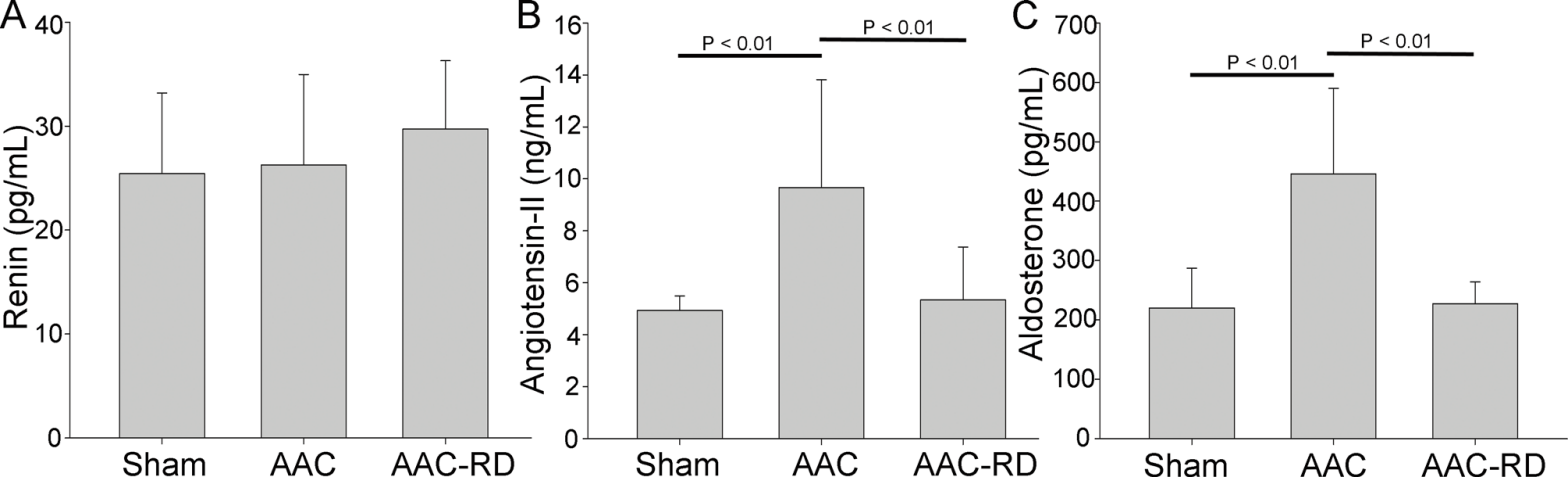
Effect of renal denervation on Renin-Angiotensin-Aldosterone System (RAAS). Plasma levels of rennin (A), angiotensin-II (B) and aldosterone (C) in the sham-operated, abdominal aortic constriction (AAC), abdominal aortic constriction with renal denervation (AAC-RD) groups.

There was no difference in plasma level of rennin among the sham-operated, AAC and AAC-RD groups (Fig 5A). Angiotensin II in the AAC group was higher than that in the sham-operated and AAC-RD groups (P<0.01) and it was indicated that AAC-mediated increase of angiotensin II could be reversed by RD (Fig 5B). Aldosterone in the AAC group was higher than that in the Sham and AAC-RD groups (P < 0.01), suggesting that RD inhibited AAC-induced aldosterone increase (Fig 5C).

### Effect of RD on protein expression of collagen, CTGF and TGF-β1 in atrial tissues

**Fig 6:**
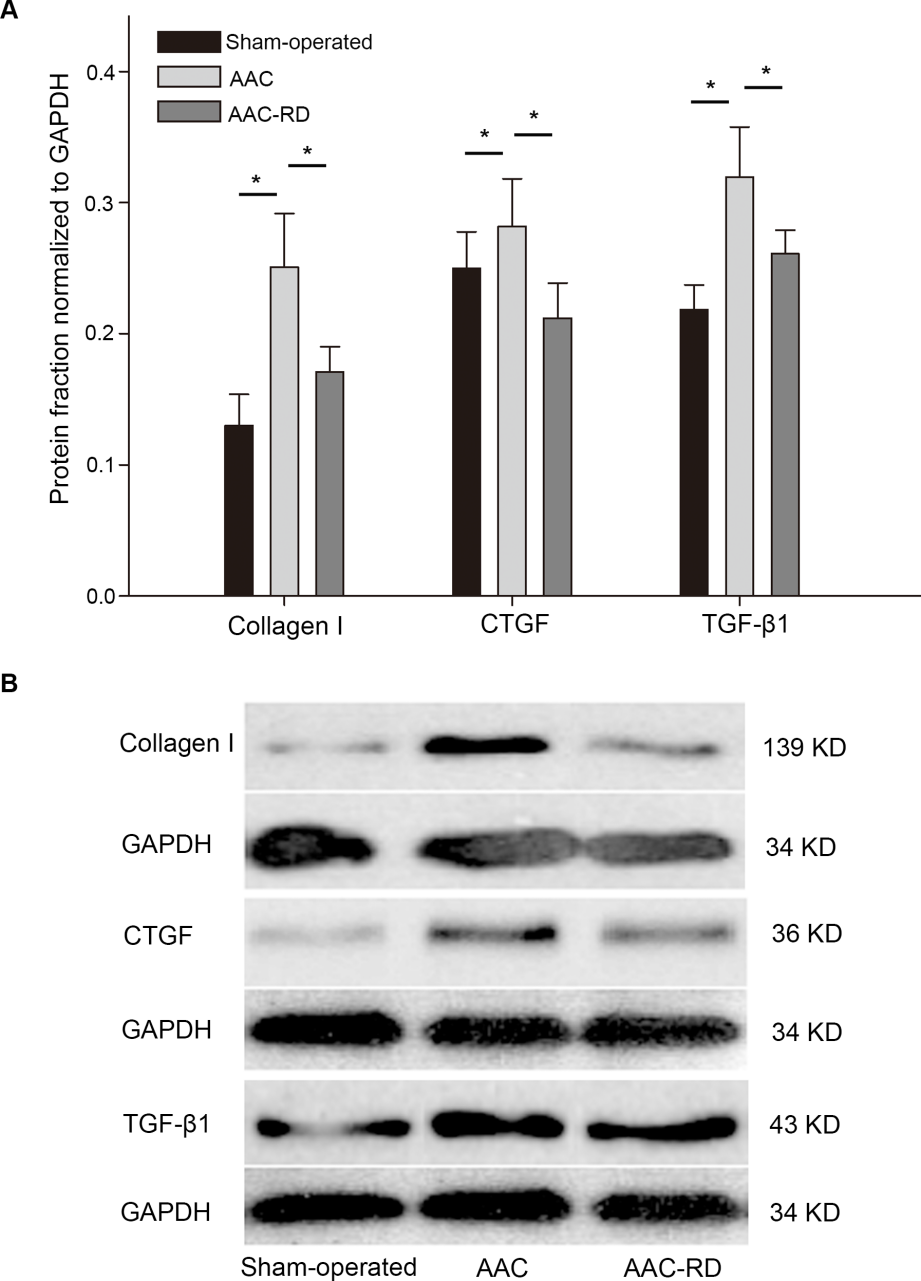
Effect of renal denervation on protein expression of collagen and profibrotic factors in atrial tissues. (A) Protein expression levels of collagen I, connective tissue growth factor (CTGF) and transforming growth factor-β1 (TGF-β1) in the Sham-operated, abdominal aortic constriction (AAC) and abdominal aortic constriction with renal denervation (AAC-RD) groups. (B) Representative images for western blotting of collagen І, TGF-β1 and CTGF. * indicated P < 0.05 between the lined two groups.

Collagen I expression was altered by AAC (Sham-operated vs. AAC, 0.13±0.02 vs. 0.25±0.04, P < 0.01), and it could be reversed by RD (AAC vs. AAC-RD, 0.25±0.04 vs. 0.17±0.02, P < 0.01) (Fig 6). CTGF and TGF-β1, two important inducers of fibrosis, were elevated by AAC, compared with the sham-operated controls. RD suppressed the AAC-induced increase of CTGF and TGF-β1 (Fig 6).

## Discussion

Recently it’s indicated that RD could inhibit myocardial fibrosis [9] and reduce atrial sympathetic nerve sprouting [10]. However, the role of RD on the progression of atrial remodeling and AF propagation has not been well investigated. In this study, we had four major findings, including 1) RD reduced the susceptibility to AF in a rabbit model of atrial fibrosis induced by AAC, 2) RD was verified to reduce atrial fibrosis and decrease RAAS activation induced by AAC, 3) RD decreased protein expression of pro-fibrotic factors as TGF-β1 and CTGF in atrial tissues, and 4) The relationship among RD, RAAS, atrial fibrosis and the susceptibility to AF was preliminary established.

### RD and sympathetic drive in AF

Although it remains controversial on the effect of catheter-based renal sympathetic denervation on resistant hypertension [11,12,13,14,15], RD might provide a new therapeutic strategy for diseases which are closely associated with hyper-sympathetic activity. Up-regulated sympathetic tone increased spontaneous triggered activity in myocardiocytes, which could subsequently induce arrhythmia [16]. Previous studies have provided evidence that the autonomic nervous system is a driver of AF [1,3]. It was highlighted that the interconnection and the crosstalk existing between the kidney and the heart included up-regulation of sympathetic nerve system, activation of the RAAS and vasopressin release. The excessive activation of the sympathetic nerve signals in the kidney was also a potential factor in the activation of the central sympathetic nerve [17]. Currently, it has been shown that modulation of the autonomic nervous system by RD could effectively reduce both renal norepinephrine spillover and whole body sympathetic activity [18,14]. RD worked as a new treatment approach to reduce sympathetic activity, blood pressure, and apnea-hypopnea index in resistant hypertension [19]. And it might simultaneously display other biological effects like anti-arrhythmia and anti-fibrosis beyond blood pressure control.

Over-activity of the sympathetic nervous system played an important role in shortening the atrial effective refractory period (AERP) [20]. It was suggested that RD reduced sympathetic nerve sprouting, inhibited pronounced shortening of the AERP and reduced susceptibility to AF in animal models of obstructive sleep apnea [21,22] by combined reduction of sympathetic drive and RAAS activity. Episodes of AF could also be eliminated by renal sympathetic denervation during short-time rapid atrial pacing [5]. Our data confirmed that RD could effectively reduce the susceptibility of AF in a rabbit model of atrial fibrosis induced by AAC.

### RD on atrial fibrosis

Taking in account to that atrial fibrosis is the most prominent manifestation of atrial structural remodeling, which plays a key role in the development and maintance of AF [23]. RD’s reduction of AF substrate might be partly attributed to fibrosis regression. Though some observations suggest that RD facilitates the regression of ventricular fibrosis [9,24], less is known about the relative impact of RD on atrial fibrosis. The excessive expression of collagen is a hallmark of fibrosis. In our animal models, atrial fibrosis was induced by pressure overload with AAC. Interestingly, we observed that RD not only altered the left but also the right atrial fibrosis induced by pressure overload. It indicated RD might play a role in anti-fibrosis beyond blood pressure. Though we didn’t directly monitor atrial pressure, but we performed echocardiography to assess left atrial diameter. For increased atrial pressure often causes atrial enlargement, none difference in left atrial diameter among the three groups indirectly indicated that AAC and RD might not alter atrial pressure in our animal model.

### RD modulated RAAS and profibrotic factors

It’s well known that the RAAS can be activated by renal sympathetic stimulation and ischemic impairment [25]. The RAAS is involved in myocardial fibrosis and increased angiotensin II production, which facillated atrial structural and electrical remodeling and created a substrate of AF [26,27,28]. This study confirmed that RD effectively attenuated AAC-inducced angiotensin II and aldsterone increase. Angiotensin II participates in the pathogenesis of heart diseases, through the regulation of inflammation and fibrosis. Type 1 receptor of angiotensin II regulates the expression of profibrotic factors as CTGF and TGF-β1 [29]. We found that RD suppressed the AAC-induced increase of CTGF and TGF-β1. On the whole, these data highlighted the complex signaling systems activated by pressure-overload and suggested a novel potential of RD to block atrial fibrosis and AF. Our data indicated AAC and RD didn’t alter plasma rennin, but AAC-induced elevation of angiotensin II could be reduced by RD. Rennin activity was characterized by the rate of angiotensinogen catalyzed by rennin to produce angiotensin I. It suggested that RD might play a role in modifying rennin activity. It was a limit for our article that rennin activity wasn’t directly determined.

Conclusion, RD reduced AF inducibility and atrial fibrosis induced by AAC, attenuated AAC-mediated angiotensin II and aldsterone increase, decreased the protein expression of CTGF and TGF-β1 in the atrium, and finally eliminated the substrate of AF. Neuro-modulation tends to be a new treatment target for arrhythmias. Catheter-based RD has the potential to improve AF prevention.

## Supporting Information

S1 Fig Effect of renal denervation on collagen fibers in the left ventricle detected by Masson staining. AAC, abdominal aortic constriction; AAC-RD, abdominal aortic constriction with renal denervation.
(TIF)
