# Supplementary figures and images for: Renal Denervation Suppresses the Inducibility of Atrial Fibrillation in a Rabbit Model for Atrial Fibrosis
Source: PLoS One. 2016 Aug 16;11(8):e0160634. doi: 10.1371/journal.pone.0160634 (PMC4986963; doi:10.1371/journal.pone.0160634)

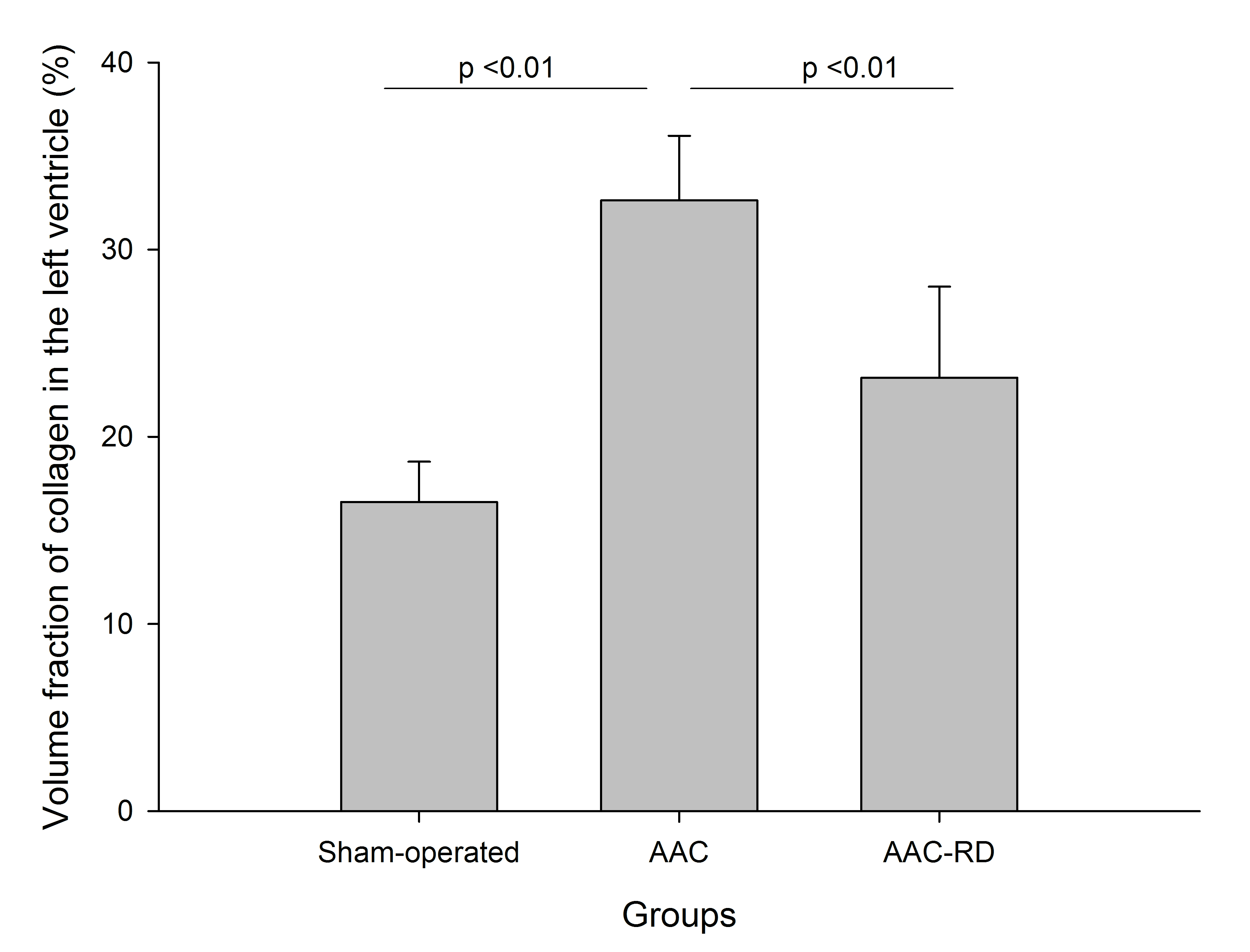

Supplement: S1 Fig — AAC, abdominal aortic constriction; AAC-RD, abdominal aortic constriction with renal denervation. (TIF) [file pone.0160634.s001.TIF]
